# Importance of the Primary Motor Cortex in Development of Human Hand/Finger Dexterity

**DOI:** 10.1093/texcom/tgaa085

**Published:** 2020-12-02

**Authors:** Eiichi Naito, Tomoyo Morita, Minoru Asada

**Affiliations:** Center for Information and Neural Networks (CiNet), National Institute of Information and Communications Technology (NICT), Suita, Osaka 565-0871, Japan; Graduate School of Frontier Biosciences, Osaka University, Suita, Osaka 565-0871, Japan; Center for Information and Neural Networks (CiNet), National Institute of Information and Communications Technology (NICT), Suita, Osaka 565-0871, Japan; Institute for Open and Transdisciplinary Research Initiatives, Osaka University, Suita, Osaka 565-0871, Japan; Center for Information and Neural Networks (CiNet), National Institute of Information and Communications Technology (NICT), Suita, Osaka 565-0871, Japan; Institute for Open and Transdisciplinary Research Initiatives, Osaka University, Suita, Osaka 565-0871, Japan

**Keywords:** deactivation, development, digital dexterity, functional magnetic resonance imaging, primary motor cortex

## Abstract

Hand/finger dexterity is well-developed in humans, and the primary motor cortex (M1) is believed to play a particularly important role in it. Here, we show that efficient recruitment of the contralateral M1 and neuronal inhibition of the ipsilateral M1 identified by simple hand motor and proprioceptive tasks are related to hand/finger dexterity and its ontogenetic development. We recruited healthy, right-handed children (*n* = 21, aged 8–11 years) and adults (*n* = 23, aged 20–26 years) and measured their brain activity using functional magnetic resonance imaging during active and passive right-hand extension–flexion tasks. We calculated individual active control-related activity (active–passive) to evaluate efficient brain activity recruitment and individual task-related deactivation (neuronal inhibition) during both tasks. Outside the scanner, participants performed 2 right-hand dexterous motor tasks, and we calculated the hand/finger dexterity index (HDI) based on their individual performance. Participants with a higher HDI exhibited less active control-related activity in the contralateral M1 defined by the active and passive tasks, independent of age. Only children with a higher HDI exhibited greater ipsilateral M1 deactivation identified by these tasks. The results imply that hand/finger dexterity can be predicted by recruitment and inhibition styles of the M1 during simple hand sensory–motor tasks.

## Introduction

Among primates, humans benefit from particularly well-developed hand/finger dexterity. The primary motor cortex (M1) is believed to play a particularly important role in hand/finger dexterity (Heffner and Masterton 1983; Isa 2019; Lemon 2019). In this study, we show that efficient recruitment of the contralateral M1 and neuronal inhibition of the ipsilateral M1 are related to hand/finger dexterity and its ontogenetic development in humans by conducting both neuroimaging and behavioral investigations.

We tested 2 major hypotheses regarding M1 involvement. First, a person with greater hand/finger dexterity tends to recruit contralateral M1 activity more efficiently in a given hand motor task. We based this hypothesis on previous studies that have shown that individuals who employ expert motor movements, like athletes and musicians, tend to recruit less activity in the contralateral M1 even when they perform a simple motor task (Naito and Hirose 2014; Callan and Naito 2014). Such efficient recruitment of contralateral M1 activity has also been reported during repeated practice of a motor experience (Picard et al. 2013) and across human development (Morita et al. 2019). Thus, efficient recruitment of contralateral M1 activity likely develops through repeated motor experiences from childhood through to adulthood, allowing us to perform skillful movements without recruiting its superfluous activity.

Second, a person with greater hand/finger dexterity develops ipsilateral M1 deactivation (inhibition) in a unimanual sensory–motor task. It has been shown in both younger and older adults that weaker interhemispheric (transcallosal) inhibition from the contralateral M1 to the ipsilateral M1 deteriorates dexterity in the right hand, as evaluated by peg task performance (Davidson and Tremblay 2013). In addition, reduced ipsilateral M1 deactivation is associated with worse peg task performance in the elderly (Loibl et al. 2011), indicating that the degree of ipsilateral M1 deactivation can be an indicator of hand/finger dexterity. Furthermore, we have recently shown that ipsilateral M1 deactivation during a unimanual right finger motor task rapidly increases from childhood to adolescence (Morita et al. 2019). Thus, ipsilateral M1 deactivation likely progresses both during childhood and after, and this progress could be associated with better performance of skillful movements.

In the neuroimaging investigation, we evaluated brain activity with functional magnetic resonance imaging (fMRI) in right-handed children (*n* = 21, aged 8–11 years) and adults (*n* = 23, aged 20–26 years) during simple active and passive right-hand extension–flexion tasks. We expected an increase in the contralateral M1 activity during the active task, which is supposed to reflect the motor control process and proprioceptive feedback processing (Amemiya and Naito 2016). Since the M1 also involves proprioceptive processing (Naito et al. 2016), we also expected an increase in the contralateral M1 activity during the passive task (Weiller et al. 1996). Since one could assume a lesser motor control component during the passive task, by subtracting the activity observed during the passive task from that of the active task, we identified active control-related activity in the M1 in addition to other motor-related regions. We used this active control-related activity as an indicator of efficient recruitment during active hand motor control. We also expected ipsilateral M1 deactivation during the active and passive tasks (Morita et al. 2019). Thus, by analyzing activity during both tasks, we could evaluate task-related deactivation, which may represent an indicator of neuronal inhibition in the ipsilateral M1 (see above). Active control-related activity and task-related deactivation were analyzed in each participant.

In the behavioral investigations outside the scanner, participants were asked to perform 2 motor tasks that require skillful manipulation of the right hand and fingers, to evaluate their hand/finger dexterity. The first was a 12-hole peg task (Henderson et al. 2007). Peg tasks have generally been used to evaluate hand/finger dexterity, especially for fingertips, across a wide participant age range (Henderson et al. 2007; Loibl et al. 2011; Davidson and Tremblay 2013). The second was a ball rotation task originally used in our previous neuroimaging studies (Kawashima et al. 1998; Uehara et al. 2011), which required continuous rotation of 2 balls in the palm of the right hand as many times as possible within an allotted time; this task can evaluate hand/finger dexterity in terms of skillful coordination of hand and finger movements. Based on these task performances, we calculated an individual hand/finger dexterity index (HDI).

We first examined whether dexterity develops from childhood to adulthood, as reported in previous studies (Dayanidhi et al. 2013; Gogola et al. 2013). Subsequently, we conducted across-task correlational analyses between the participants’ HDI and brain activity index data (active control-related activity and task-related deactivation) to examine whether greater hand/finger dexterity is correlated with a reduction in active control-related activity of the contralateral M1 and with greater deactivation in the ipsilateral M1.

## Materials and Methods

### Participants

Altogether, 44 healthy, right-handed children and adults participated in the study. The child group (CH group) consisted of 21 children (12 males; mean age, 9.5 ± 1.0 years; range, 8 years and 8 months to 11 years and 10 months). The adult group (AD group) consisted of 23 young adults (11 males; mean age, 22.1 ± 1.4 years; range, 20 years and 3 months to 26 years and 3 months). The children and adults were recruited from local elementary schools and universities. Data were excluded from 3 other children because of excessive head movement during fMRI scanning (see below). We confirmed participants’ handedness with the Edinburgh Handedness Inventory (Oldfield 1971) and ensured that no participants had a history of neurological or psychiatric disorder, per self-reports, and reports provided by legal guardians.

The study protocol was approved by the Ethics Committee of the National Institute of Information and Communications Technology. We explained the details of the study to the participants before the start of the experiment, after which the participants provided written informed consent. Written informed consent was also obtained from the children’s legal guardians. The study was carried out according to the principles and guidelines of the Declaration of Helsinki (1975).

### Motor Tasks to Evaluate Hand/Finger Dexterity

**Figure 1:**
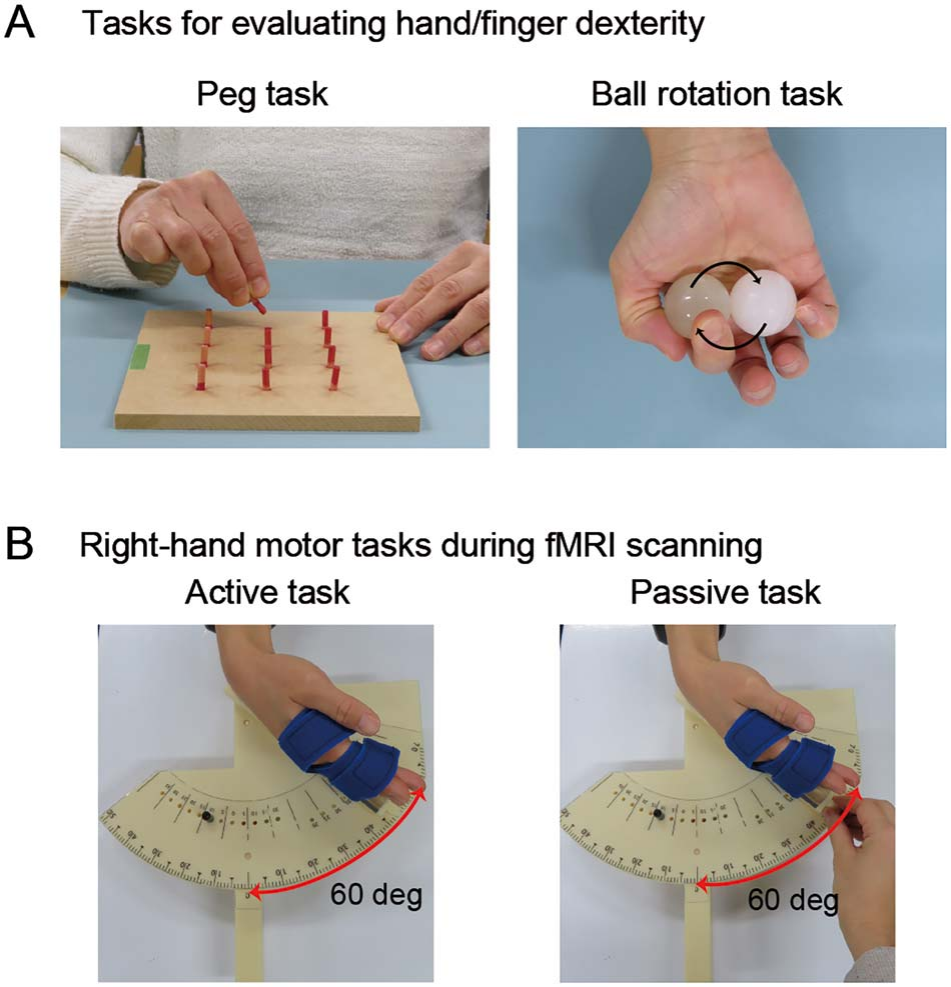
Right-hand motor tasks. (*A*): The 12-hole peg (left) and ball rotation (right) tasks used to evaluate hand/finger dexterity. Green tape on the peg board indicates the hand’s starting position. (*B*): The active (left) and passive (right) motor tasks performed during the fMRI scans. For these tasks, please see also Supplementary Material.

We used the 12-hole peg task and an original ball rotation task (Fig. 1*A*), which require skillful manipulation of the right hand and fingers to evaluate hand/finger dexterity. These behavioral evaluations were carried out prior to the fMRI scanning.

#### Twelve-Hole Peg Task

During the 12-hole peg task, the participants had to repeatedly remove a small peg inserted in 1 of 12 holes on a board with their right fingers, vertically flip the peg, and reinsert the peg into the same hole. In this task, a participant who is more able to rapidly coordinate their fingertip movements without generating superfluous movements should be able to complete the task quickly. We asked the participants to complete this task as fast as they possibly could. We measured the time required to flip all 12 pegs with a stopwatch. Since none of our participants had previously experienced this task, each participant performed the peg task 3 times. We used the individual best (shortest) time for the 3 trials in the analysis and performed a *t*-test to evaluate for between-group differences.

#### Ball Rotation Task

The participants continuously rotated 2 balls (diameter, 30 mm; 45 g each) clockwise in the palm of their right hand as many times as possible for 15 s. We video recorded the movements and counted the number of rotations via off-line visual inspection. In our preliminary study, we tested several sizes of balls and confirmed that children were able to rotate them. We measured the size of the hand (from the tip of the middle finger to the bottom of the palm) in each participant and confirmed that hand size was not correlated with the HDI orthogonalized by age (*r* = 0.05), though it was correlated with age (*r* = 0.77).

The ball rotation task requires skillful coordination of hand and finger movements (Uehara et al. 2011), with little verbal cognitive aid. In this task, a participant who is more able to rapidly coordinate their hand and finger movements without generating superfluous movements should be able to perform more ball rotations during the 15 s. None of the participants had experienced this task before the present study. Our previous study demonstrated that this task is complex, but that performance improves within several trials, indicating a learning effect (Kawashima et al. 1998). Thus, each participant performed this task 10 times (trials). We analyzed the average individual number of rotations across the 10 trials of this task, since the average was highly correlated with the best performance across 10 trials, the performance of the first trial, and the performance of the last trial across participants (*r* = 0.99, 0.95, 0.96, respectively).

To illustrate the learning effect, we calculated a performance improvement ratio from the 1st to the 10th trial for each participant, by dividing the number of rotations in the last trial by the number in the first trial. We then calculated the average of this across participants in each group and performed a *t*-test to evaluate for between-group differences.

### Evaluation of Hand/Finger Dexterity

**Figure 2:**
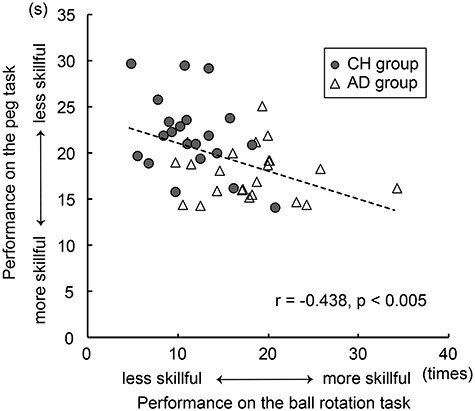
Correlation between performance in the 12-hole peg and ball rotation tasks across participants. The dashed line represents the linear regression line fitted to the data. The horizontal (*x*) axis indicates the number of ball rotations, and the vertical (*y*) axis indicates the time to flip all of the pegs (s). These were significantly negatively correlated with each other (*r*_44_ = −0.44, *P* < 0.005). The adults exhibited greater dexterity in both tasks.

We examined the correlation between performances in the 2 dexterous motor tasks across all participants (Fig. 2). We then calculated the average time required to complete the peg task and the average number of ball rotations in each group and performed a *t*-test to evaluate for between-group performance differences in each task. We calculated individual HDI scores based on the 2 motor performances. We first calculated a *z*-score of individual performance in each task. This individual *z*-score was computed based on the mean and its standard deviation across all 44 participants. The sign of the *z*-score obtained from the peg task was reversed, so that higher *z*-scores represented better performance. We then calculated the individual HDI by averaging the 2 *z*-scores obtained from the 2 tasks.

### fMRI Tasks

We used fMRI to assess brain activity during the active and passive right-hand extension–flexion tasks (Fig. 1*B*), which have been previously described (Amemiya and Naito 2016). The participant’s right hand was fixed to a plastic apparatus. A mobile indicator was mounted on the surface of this apparatus, and angular degrees were scaled using an ordinal protractor. We fixed the right hand to the mobile indicator with 2 hook-and-loop fasteners. The index, middle, ring, and little fingers were extended. Special care was taken to ensure that the 2 fasteners were consistently wrapped around the hand of each participant to match the areas that received tactile inputs from the fasteners. One fastener was wrapped around the proximal interphalangeal joints, and the other was wrapped around the metacarpal bones. The radiocarpal joint of the wrist was located immediately above the beginning of the protractor. We defined the wrist angle as 0° when the wrist was straightened in the start position.

#### Active Task

The participants were blindfolded and asked to continuously exert cyclic extension–flexion movements of their right wrists in precise synchronization with 1-Hz audio tones generated by a computer (see Supplementary Fig. 1). We fixed 2 stoppers onto the protractor device to control the range of wrist motion across fMRI epochs and participants (Fig. 1*B*). One was fixed to the start position to prevent the wrist from extending beyond the straight (0°) position, whereas the other was positioned to prevent the wrist from flexing beyond 60°. The participants had to touch either stopper (0° or 60°) with the hand-fixed mobile indicator in precise synchronization with the 1-Hz audio tones while making controlled alternating wrist extension–flexion movements (see Supplementary Video 1). The movements were visually inspected by an experimenter who stood beside the scanner bed.

#### Passive Task

The blindfolded participants were then asked to passively experience extension–flexion of their right wrist in an expert-guided manner (see Supplementary Fig. 1); the experimenter (E.N.) moved the hand-fixed mobile indicator in the 0°–60° motion range. The experimenter continuously controlled alternating wrist extension–flexion movements between the straight (0°) position and the 60° flexion position to touch either stopper (0° or 60°) with the hand-fixed mobile indicator in precise synchronization with the 1-Hz audio tones (see Supplementary Video 2). We asked the participant to relax their hands, not to generate the movements by themselves, and not to resist the passive movements. Indeed, additional electromyogram (EMG) assessments during the passive task, which were conducted later, outside the scanner, implied that participants were unlikely to actively generate the movements or resist the movements during the passive task (see Supplementary Material and Supplementary Fig. 2).

#### fMRI Task Procedure

Before the fMRI experiment, each participant experienced the active and passive tasks outside the magnetic resonance imaging (MRI) room to familiarize them with the tasks. The participants then entered the room and laid down in the MRI scanner. Their heads were immobilized using sponge cushions and adhesive tape, and their ears were plugged. Their left and right arms were naturally semipronated and extended in front of them. Both arms were supported by cushions, allowing the participants to relax their upper arms during the tasks. We asked the participants to relax their entire body, to refrain from producing unnecessary movements, and to only think of the assigned tasks.

The active and passive tasks were performed in an alternating manner, with half of the participants starting with the active task. Each participant completed 2 experimental 205-s runs for each task. One run comprised 6 task epochs, each of which lasted for 15 s. The task epochs were separated by 15-s baseline (rest) periods. We asked participants to close their eyes before starting each run. During the run, we provided the participants with auditory instructions that indicated the start of a task epoch (e.g., 3, 2, 1, start) through a magnetic resonance-compatible headphone. We also provided a “stop” instruction generated by a computer to notify the participants of the end of each epoch. The 1-Hz audio tones were also generated during the rest periods. The participants heard the 1-Hz auditory stimuli but did not move their right hand during the rest periods. Each run also included a 25-s baseline period before the start of the first epoch and another 15-s baseline period after the end of the final epoch.

### fMRI Data Acquisition

The fMRI images were acquired using *T*_2_^*^-weighted gradient echo-planar imaging (EPI) sequences with a 3.0-Tesla MRI scanner (Trio Tim; Siemens Healthineers) and a 32-channel array coil. Each volume consisted of 44 slices (slice thickness = 3.0 mm, interslice thickness = 0.5 mm) acquired in ascending order, covering the entire brain. The interval between successive acquisitions from the same slice lasted 2500 ms. An echo time of 30 ms and a flip angle of 80̊ were used. The field of view was 192 mm × 192 mm, and the matrix size was 64 × 64. Voxel dimensions were 3 mm × 3 mm × 3.5 mm in the *x*-, *y*-, and *z*-axes, respectively. We collected 82 volumes in each experimental run.

### fMRI Data Preprocessing

To eliminate the effects of unsteady magnetization during the tasks, we discarded the first 4 EPI images in each fMRI run before the first epoch started. Imaging data were analyzed using SPM 12 (Wellcome Trust Centre for Neuroimaging, London, UK) implemented in Matlab (Mathworks).

The present study was a developmental fMRI study, in which greater head motion in children is often a matter of concern. Therefore, we took special care regarding head motion in the analyses (Morita et al. 2019). EPI images were realigned to the first image. Through this realignment procedure, we obtained head position data that changed over time from the first frame through 6 parameters (translational displacements along *x*-, *y*-, and *z*-axes, and the rotational displacements of pitch, raw, and roll). Then, we calculated the absolute value of displacement in each frame relative to its previous frame (framewise displacement [FD]; Power et al. 2012). This was done for every translational and rotational axis. We totaled these values per frame. In this calculation, we converted the rotational displacements from degrees to millimeters by calculating the displacement on the surface of a sphere with a 50-mm radius, which is approximately the mean distance from the cerebral cortex to the center of the head (Power et al. 2012).

To check the change in FD values through all frames of an entire experimental run, we used previously published guidelines and counted the number of frames for each participant that had an FD over 0.9 mm (Siegel et al. 2014; Morita et al. 2019). Three children who had more than 10 frames (3%) across the 4 runs with FD values greater than 0.9 mm were excluded from the data analysis. The number of frames in which FD exceeded 0.9 mm was zero in most participants (CH group, 12/21; AD group, 23/23). The average FD of all frames across participants was 0.095 ± 0.044 and 0.065 ± 0.022 mm for the CH and AD groups, respectively. These values were much smaller than previously reported values (Engelhardt et al. 2017), possibly due to our careful prevention of head motion during scanning. The FD during the task epoch was smaller than during the baseline (rest) period, regardless of age group or task, similar to previous studies (Engelhardt et al. 2017; Morita et al. 2019).

The realigned images were normalized to the Montreal Neurological Institute (MNI) space (Evans et al. 1994). The fMRI data analysis obtained from children and adults within a common MNI space has been previously validated (Kang et al. 2003). Finally, the spatially normalized fMRI images were filtered using a Gaussian kernel with a full width at half maximum of 4 mm along the *x*-, *y*-, and *z*-axes.

### Single-Subject Analysis

Following preprocessing, we used a general linear model (Friston et al. 1995; Worsley and Friston 1995) to analyze the fMRI data. In the single-subject analysis, the design matrix contained a boxcar function for the task epoch in each run (active or passive), which was convolved with a canonical hemodynamic response function. To correct for residual motion-related variance after realignment, the 6 realignment parameters were also included in the design matrix as regressors of no interest.

We generated an individual image showing active control-related activity by subtracting activity during the passive task from activity during the active task (active–passive) in each participant. These individual images were used in the following random effects group analysis and multiple regression analysis. We also generated an individual image showing task-related deactivation by averaging the data obtained from the active and passive tasks, using it in the following multiple regression analysis. We averaged the data because ipsilateral M1 deactivation has been reported not only during active hand movement (Morita et al. 2019 and more) but also during passive movement (Petsas et al. 2013), such that we could expect a similar deactivation in the ipsilateral M1 during both the active and passive tasks. We also generated an individual image showing a task-related deactivation pattern for each task in each participant, using it in the following conjunction analysis.

### Identification of Active Control-Related Activity in Random Effects Group Analysis

**Figure 3:**
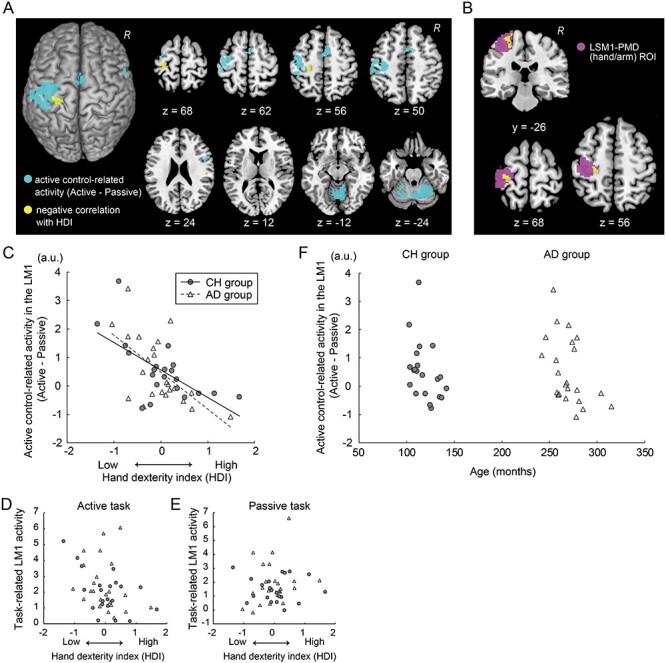
Active control-related activity and its relationship with hand/finger dexterity. (*A*): Brain regions that showed active control-related activity (light blue) identified in the random effects group analysis. The yellow section indicates the largest voxel cluster where active control-related activity was negatively correlated with the HDI across all participants. These images were superimposed on the MNI standard brain. The ipsilateral hemisphere is on the right. (*B*): The yellow cluster indicates a significant cluster in the hand/arm section of the contralateral SM1-PMD ROI (pink). Data from a coronal section (*y* = −26) and horizontal section (*z* = + 68 and 56) are shown. (*C*): Relationship between the HDI orthogonalized by age and active control-related activity in the contralateral M1 in each age group. Horizontal (*x*) axis indicates the HDI. Vertical (*y*) axis indicates effect size of active control-related activity (a.u.). Filled dots represent individual data obtained from the CH group, and open triangles indicate those from the AD group (also in panels *D*, *E*). The dashed line indicates a regression line fitted to the data from the AD group, and a solid line indicates a regression line fitted to the data from the CH group. (*D*): Relationship between the HDI orthogonalized by age (*x*-axis) and task-related activity in the contralateral M1 during the active task (*y*-axis) in each age group. (*E*): Relationship between the HDI orthogonalized by age (*x*-axis) and task-related activity in the contralateral M1 during the passive task (*y*-axis) in each age group. (*F*): Relationship between age and active control-related activity in the contralateral M1 in each age group. Horizontal (*x*) axis indicates age (in months). Abbreviations: a.u., arbitrary unit; LM1, left M1; R, right hemisphere; SM1, primary sensorimotor cortex.

To illustrate brain regions that show active control-related activity in the entire brain (Fig. 3*A*), we examined active control-related activity at the group level by performing a random effects group analysis (Holmes and Friston 1998) using the images obtained from all 44 participants. We reported this group-level result using the family-wise error rate (FWE)-corrected extent threshold of *P* < 0.05 across the entire brain for a voxel-cluster image generated at the uncorrected height threshold of *P* < 0.005. To identify the anatomical regions corresponding to the active control-related activity peaks, we referenced the cytoarchitectonic probability maps from the MNI standard brain in the SPM Anatomy Toolbox v2.2b (Eickhoff et al. 2005), which was also used in the following analyses. We defined the primary motor cortex (M1) based on cytoarchitectonic probability maps of areas 4a and 4p, and the primary sensorimotor cortices (SM1) based on cytoarchitectonic probability maps of areas 4a, 4p, 3a, 3b, and 1.

### Relationship Between Active Control-Related Activity and Hand/Finger Dexterity

To test our hypothesis that reduced active control-related activity in the contralateral M1 is correlated with greater hand/finger dexterity, we performed a multiple regression analysis using individual images showing active control-related activity. In this analysis, we included 2 regressors. One was a regressor of age (in months) of all participants, and the other was a regressor of the HDI (see above) of all participants, which was orthogonalized by age. We applied Gram–Schmidt orthogonalization to the HDI using the “spm_orth.m” function in the SPM toolbox. This allowed us to identify brain regions in which active control-related activity was purely correlated with hand/finger dexterity independent of age, since original hand/finger dexterity data were correlated with age. We also conducted the same regression analysis in each group separately.

We generated a voxel-cluster image with an uncorrected height threshold of *P <* 0.005. We first examined significant clusters using the FWE-corrected extent threshold of *P* < 0.05 across the entire brain. We also performed region of interest (ROI) analysis (using a small volume correction [SVC] approach) for the contralateral M1, since we had a strong a priori anatomical hypothesis for the M1 (see section Introduction). The ROI image was prepared based on our previous independent fMRI study (Naito et al. 2007), where we vibrated the tendon of the right hand and 19 participants experienced illusory movement of their right hand. In this study, we reported an active-voxel cluster (8208 mm^3^) in the hand/arm section of the contralateral primary sensorimotor cortices (SM1) including the dorsal premotor cortex (PMD; Fig. 3*B*). We used this cluster image as an ROI image for the contralateral SM1-PMD (hand/arm section) and used an FWE-corrected extent threshold of *P* < 0.05 (SVC in the ROI; Worsley et al. 1996).

To ensure the validity of our findings, we plotted the individual effect size of active control-related activity against the individual HDIs orthogonalized by age (Fig. 3*C*) and against individual age (Fig. 3*F*) in the CH and AD groups separately. For each participant, we extracted the effect size of active control-related activity from a 4-mm (radius) sphere around the left M1 peak (MNI coordinates: *x*, *y*, *z* = −28, −28, 68), which was identified in the above multiple regression analysis performed for all participants’ data. We also extracted the effect size of task-related activity in this region during the active and passive tasks in each participant to evaluate the relationship between the individual effect size of the task-related activity and the individual HDIs orthogonalized by age (Fig. 3*D*,*E*). These analyses were done purely for visualization purposes; we did not perform any statistical evaluations, in order to avoid the circular evaluation issue raised by Kriegeskorte et al. (2009).

### Relationship Between Task-Related Deactivation and Hand/Finger Dexterity

We also tested another hypothesis that greater task-related deactivation in the ipsilateral M1 is correlated with greater hand/finger dexterity. We performed a multiple regression analysis using the individual images showing task-related deactivation during both the active and passive tasks (see above). In this analysis, we included the same 2 regressors (age and HDI orthogonalized by age). We also conducted the same regression analysis in each age group separately.

**Figure 4:**
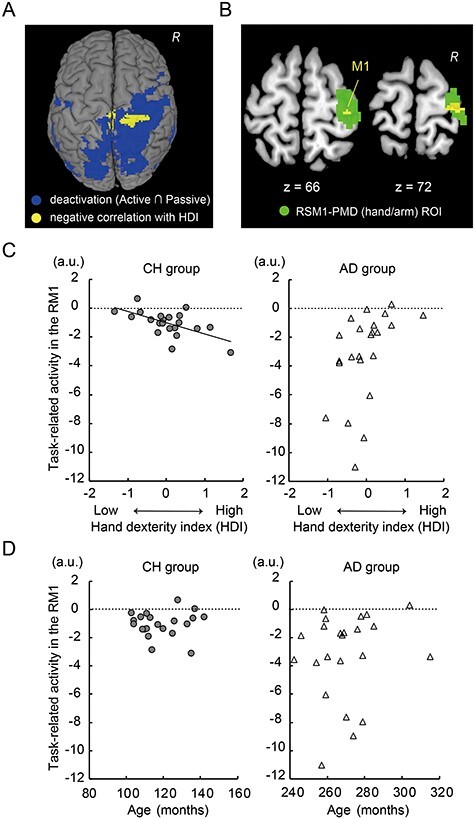
Task-related deactivation and its relationship with hand/finger dexterity. (*A*): Brain regions consistently deactivated during the active and passive tasks across all participants (blue). There were 2 significant voxel clusters in which task-related deactivation was correlated with the HDI (yellow), but only in the CH group. Images were superimposed onto the MNI standard brain template. The yellow sections are located in the hand/arm and trunk sections of the ipsilateral M1 and in the foot section of the contralateral M1. (*B*): The yellow cluster formed a significant cluster in the hand/arm section of the ipsilateral SM1-PMD ROI (green). Data from a horizontal section (*z* = + 66 and 72) are shown. (*C*): Relationship between the HDI orthogonalized by age and task-related activity in the ipsilateral M1 (left panel for CH group and right panel for AD group). Horizontal (*x*) axis indicates the HDI. Vertical (*y*) axis (also in panel *D*) indicates the effect size of task-related activity (a.u.: negative value indicates deactivation). The dashed line in the left panel indicates a regression line fitted to the data from CH group. (*D*): Relationship between age and task-related activity in the ipsilateral M1 (left panel for the CH group and right panel for the AD group). The horizontal (*x*) axis indicates age (in months). Abbreviations: RM1, right M1.

We generated a voxel-cluster image with an uncorrected height threshold of *P <* 0.005. We first examined significant clusters using the FWE-corrected extent threshold of *P* < 0.05 across the entire brain. We also performed a similar ROI analysis for the ipsilateral M1, based on a strong a priori anatomical hypothesis for the M1 (see Introduction). This ROI image was also prepared based on the previously described fMRI study (Naito et al. 2007). In this study, we reported an active-voxel cluster (10 125 mm^3^) in the hand/arm section of the ipsilateral SM1 including PMD (Fig. 4*B*). We used this cluster image as an ROI image for the ipsilateral SM1-PMD (hand/arm section) and used an FWE-corrected extent threshold of *P* < 0.05 (SVC in the ROI).

To validate our findings, we plotted the individual effect size of task-related activity against the individual HDI orthogonalized by age (Fig. 4*C*) and against the individual age (Fig. 4*D*) for the CH and AD groups separately. In each participant, we extracted the effect size of task-related activity from a 4-mm sphere around the right M1 peak (MNI coordinates: *x*, *y*, *z* = 36, −26, 66), which was identified in the above multiple regression analysis performed for the children’s data. These analyses were done purely for visualization purposes.

### Identification of Consistent Task-Related Deactivation Between Active and Passive Tasks in the Conjunction Analysis

The conjunction analysis served to demonstrate that the significant cluster of active voxels (correlation result; Fig. 4*A* yellow section) is located in the brain regions that were consistently deactivated during both the active and passive tasks. We performed the conjunction analysis (Price and Friston 1997) to identify brain regions showing similar patterns of task-related deactivations between the 2 tasks (active < baseline ∩ passive < baseline). We also report the result using a FWE-corrected extent threshold of *P* < 0.05 across the entire brain for a voxel-cluster image generated at the uncorrected height threshold of *P* < 0.005.

## Results

### Dexterous Motor Performance Correlation and Group Differences

The correlation analysis between the 2 dexterous motor performances across all participants revealed a significant negative correlation between the time required to complete the peg task and the number of ball rotations (*r*_44_ = −0.44, *P* < 0.005; Fig. 2), indicating that performance in these 2 tasks is not independent when we analyzed all participants’ data.

Although some children performed as well as adults, the adult participants generally showed greater dexterity in both tasks (Fig. 2). The average time required to complete the peg task was 17.9 ± 2.8 and 22.0 ± 4.2 s in the AD and CH groups, respectively. The AD group required significantly less time than the CH group (*t*_42_ = 3.83, *P* < 0.001). Likewise, the average number of ball rotations was 18.0 ± 5.6 and 11.5 ± 4.1 times in the AD and CH groups, respectively. The AD group performed a significantly greater number of ball rotations than the CH group (*t*_42_ = 4.42, *P* < 0.001). Indeed, the HDI calculated using the 2 dexterous motor tasks was significantly correlated with age (*r*_44_ = 0.69, *P* < 0.001). These results support the notion that human hand/finger dexterity develops from childhood to adulthood, as reported in previous studies (Dayanidhi et al. 2013; Gogola et al. 2013).

In the ball rotation task, we found a gradual improvement in performance from the 1st to the 10th trial in both age groups. The improvement ratio was not significantly different between the 2 groups (AD group, 1.16 ± 0.23; CH group, 1.15 ± 0.30).

### Reduced Active Control-Related Activity in the Contralateral M1 Is Associated With Greater Hand/Finger Dexterity

The random effects group analysis revealed an increase in active control-related activity (active > passive) in the contralateral SM1 (peaks in cytoarchitectonic areas 4a, 4p, 3b, and 1), PMD (area 6), bilateral supplementary motor areas (SMAs; area 6), right inferior frontal cortex (area 44), and bilateral cerebellum (Fig. 3*A*). Thus, an increase in active control-related activity of the contralateral M1 was the group effect in all present participants.

Among these brain regions, we found the largest cluster in the hand/arm section of the contralateral SM1-PMD (cluster size = 97), in which active control-related activity was negatively correlated with the HDI (orthogonalized by age) across all participants. This cluster was significant in the contralateral SM1-PMD hand/arm ROI (82 voxels; *P* < 0.01 after SVC; Fig. 3*B*). Correlated activity peaks were located in the cytoarchitectonic areas 4a (MNI coordinates: *x*, *y*, *z* = −28, −28, 68) and 4p (−24, −26, 56). The 4a voxel showed the highest correlation with the HDI across the entire brain (*T* = 5.30).

We found no regions in which active control-related activity was positively correlated with the HDI, either at the level of the entire brain or the ROI. Similarly, no regions showed significant negative correlations with the HDI even in the ROI when task-related activity from only the active or passive task was used.

When we separately examined brain regions with negative correlations in the CH and AD groups, we found a small cluster of active voxels (height threshold, *P* < 0.005) closely located at the 4a peak in both groups (data not shown). We found a cluster of 11 active voxels (not significant) in both groups (AD peak coordinates = −32, −24, 68, *T* = 3.89; CH peak coordinates = −28, −28, 70, *T* = 3.83).

The validity of these findings was supported when we plotted the individual effect size of active control-related activity against the individual HDI orthogonalized by age (Fig. 3*C*). We found a highly negative correlation between the effect size of active control-related activity around the contralateral 4a peak (−28, −28, 68) and the HDI across participants in both groups (all: *r*_44_ = −0.58; CH: *r*_21_ = −0.64; AD: *r*_23_ = −0.56). As described above, neither in the active task nor in the passive task was such a high correlation observed when we examined correlations between task-related activity in this region and the HDI across participants (Fig. 3*D*,*E*). This indicated that the active control-related activity (active–passive) is a better indicator for dexterity than the mere task-related activity. However, it was also true that we observed a moderately negative correlation between the task-related activity in the active task and the HDI across all participants (all: *r*_44_ = −0.38; Fig. 3*D*), whereas no correlation was observed in the passive task (all: *r*_44_ = 0.09; Fig. 3*E*). This indicated that the negative correlation observed in the active task mainly contributes to the correlation between the active control-related activity and the HDI (Fig. 3*C*). Finally, the effect size of active control-related activity was not correlated with age per se (Fig. 3*F*).

These results indicate that, regardless of age group, participants with greater hand/finger dexterity tend to show less active control-related activity in the hand/arm section of the contralateral M1.

### Greater Deactivation in the Ipsilateral M1 Is Associated With Greater Hand/Finger Dexterity During Childhood

Unlike the active control-related activity, we did not find any regions in which the degree of task-related deactivation during the active and passive tasks was correlated with the HDI in all participants. Instead, we found 2 significant voxel clusters in the CH group in which the degree of task-related deactivation was correlated with the HDI in the ipsilateral SM1-PMD (FWE-corrected extent threshold of *P* < 0.05 across the entire brain). One was located in the hand/arm and trunk sections of the ipsilateral SM1-PMD (cluster size = 239; trunk peak coordinates = 12, −28, 70), and the other was in the foot section of the contralateral M1 (cluster size = 126; peak coordinates = −2, −20, 68). These clusters were located within the regions consistently deactivated between the active and passive tasks revealed by the conjunction analysis (Fig. 4*A*). In the ipsilateral SM1-PMD hand/arm ROI, we found a significant cluster of active voxels (81 voxels, *P* < 0.01 after SVC, Fig. 4*B*). The correlated activity peak (36, −26, 66) was located in the cytoarchitectonic area 4a (*T* = 4.79).

The validity of these findings was supported when we plotted the individual effect size of the task-related activity against the individual HDI orthogonalized by age (Fig. 4*C*). There was a negative correlation between the effect size of task-related activity obtained from the right area 4a peak (36, −26, 66) and the HDI in the CH group only (*r*_21_ = −0.60). We also confirmed that the effect size of task-related activity was not correlated with age per se (*r*_21_ = −0.06, Fig. 4*D*). Hence, children with greater hand/finger dexterity tended to show greater deactivation in the ipsilateral M1, but adults did not.

## Discussion

We demonstrated that participants with greater hand/finger dexterity tend to show less active control-related activity in the contralateral M1, as defined by simple hand motor and proprioceptive tasks, independent of age. In addition, we showed that only children with greater dexterity tend to show greater ipsilateral M1 deactivation during these tasks. These results imply that hand/finger dexterity from childhood through to adulthood can be predicted by efficient recruitment of the contralateral M1 activity during a simple hand motor task and that dexterity during childhood can be predicted by the degree of deactivation (inhibition) in the ipsilateral M1 during simple unimanual sensory–motor tasks.

### Relationship Between Contralateral M1 Activity During the Passive and Active Tasks

The random effects group analysis revealed an increase of active control-related activity (active > passive) across the broader sensorimotor regions, including the contralateral M1 (Fig. 3*A*). This finding fits with those of a previous report (Mima et al. 1999). In the present study, we used active (motor) and passive (proprioceptive) tasks, in which we visually checked that all participants either performed or experienced alternating wrist extension–flexion movements in a 60-degree range of motion in synchronization with 1-Hz audio tones (see also Supplementary Fig. 1). We expected an increase of active control-related activity in the contralateral M1 in all participants.

As we expected, we observed a robust increase of active control-related activity in the contralateral M1 region (the sphere around the peak [−28, −28, 68]) in 26 participants (CH group: *n* = 14; AD group: *n* = 12; Fig. 3*C*). In contrast, we could not observe a clear increase in active control-related activity in this region in the 18 remaining participants (CH group: *n* = 7; AD group: *n* = 11; Fig. 3*C*). Upon assessing this increase, we found a significant cluster of voxels (629 voxels; MNI coordinates of a peak voxel [area 4a] = −40, −16, 56) in the contralateral (left) SM1-PMD ROI when we contrasted the active with the passive task in the 26 participants. Conversely, in the 18 participants, no voxels and only 2 voxels were identified in this ROI when we contrasted the passive with the active task, and the active with the passive task, respectively. This means that even though activity around the contralateral M1 region (−28, −28, 68) appeared to be slightly greater during the passive task than the active task in these 18 participants (Supplementary Material and Supplementary Fig. 3), the increase was not significant and their contralateral (left) M1 activity was comparable between the active and passive tasks. This indicates that, in the active task, the M1 of these participants could somehow generate movements merely by the same amount of blood oxygenation level-dependent (BOLD) signal (=synaptic activity) as the M1 showed in the passive task.

When we further assessed the characteristics of task-related activity during the passive task in the 18 participants (Supplementary Material and Supplementary Fig. 4), we found that, in the entire brain, the sensorimotor network of area 2, caudal cingulate motor area, and cerebellar vermis, which all likely receive proprioceptive input and engage proprioceptive processing (Jueptner et al. 1997; Naito et al. 2005, 2016), were significantly more activated during the passive task, as compared with the 26 participants. The lack of contralateral M1 activity in this comparison seems to rebuff the notion that these 18 participants generated a greater amount of active or resistant motor components during the passive task than the 26 participants. The putative lack of any active or resistant motor components during the passive task does not contradict the EMG assessment results (Supplementary Material and Supplementary Fig. 2). The sensorimotor network of the 18 participants might receive a greater amount of proprioceptive input during the passive task, possibly due to a higher sensory gain during the task, even though all participants experienced the same proprioceptive task.

Taken together, these results may imply that the 18 participants efficiently recruited the contralateral M1 activity while controlling proprioceptive input during the active task. This efficient control could be possible if these participants made use of a sensorimotor transformation (integration) function implemented in the contralateral M1 (Cheney and Fetz 1984; Pruszynski et al. 2011; Naito et al. 2016) during the active task by flexibly controlling sensory gain simultaneously (e.g., Seki and Fetz 2012; Confais et al. 2017). Although the lack of a precise evaluation of hand movements and muscle activity during the active and passive tasks in the scanner makes drawing conclusions difficult, the results imply that people who can efficiently recruit the contralateral M1 by flexibly controlling sensory gain have better hand/finger dexterity. Further investigations are warranted to explore the underlying neuronal substrates of the finding.

### Active Control-Related Activity and Hand/Finger Dexterity

In the present study, we did not directly measure brain activity during the performance of the 2 dexterous motor tasks, since these tasks are very complex and it is difficult to perform passive tasks to identify their active control-related activity in a reliable manner. Using simple movement presents the advantage of being more precisely controlled passively than dexterous movement. In addition, there might be smaller individual differences in terms of movement strategy, kinematics, and dynamics in a simple movement than in a dexterous movement.

Even though we used the independent, simple motor task to evaluate active control-related activity, we may still evaluate the individual difference in how a person recruits contralateral M1 activity when they actively control their right hand in general. The current work focused on explaining individual hand/finger dexterity by individual brain activity during performance of an independent and simple task, but the across-task correlation results appear reproducible. In an independent fMRI study in which we conducted a similar experiment in another group of healthy, right-handed adults (*n* = 32, aged 25–59 years), we confirmed that active control-related activity in the contralateral M1 was negatively correlated with hand/finger dexterity evaluated by the 12-hole peg task (see Supplementary Material).

Among the various sensorimotor regions that showed an increase in active control-related activity (Fig. 3*A*), the largest voxel cluster with activity that was strongly negatively correlated with the HDI was identified in the hand/arm section of the contralateral SM1-PMD (peaks in areas 4a and 4p; Fig. 3*A*,*B*). This suggests that within the whole brain, efficient recruitment of contralateral M1 activity is particularly important for hand/finger dexterity. The M1 activity was correlated with the HDI in both age groups (Fig. 3*C*), but not with age per se (Fig. 3*F*). This indicates that efficient recruitment of the contralateral M1 activity is a consistent key factor for greater hand/finger dexterity throughout childhood and into adulthood.

We also confirmed that the active control-related activity (active–passive) is a better indicator for dexterity than the mere task-related activity during the active or passive task, regardless of age (Fig. 3*C*–*E*), though task-related activity during the active task was likely a major contributor (Fig. 3*D*,*E*). In the 26 CH and AD participants who showed significantly greater task-related activity during the active task than during the passive task (see above), the contralateral M1 activity was additionally recruited during the active task relative to the passive task (Supplementary Fig. 3), and greater dexterity was observed in the participants who efficiently recruited less additional activity in the contralateral M1 (Fig. 3*C*). Similarly, as discussed above, the 18 remaining CH and AD participants likely recruited the contralateral M1 activity very efficiently while controlling proprioceptive input during the active task, and they generally showed greater dexterity than the 26 participants (Fig. 3*C*). Taken together, these results imply that participants with greater hand/finger dexterity tend to efficiently recruit activity in the hand/arm section of the contralateral M1 during active hand motor control in general, regardless of age.

### Ipsilateral M1 Deactivation

The physiological mechanisms underlying the task-induced negative BOLD phenomenon, indicative of deactivation, are not fully understood (Kim and Ogawa 2012; Moraschi et al. 2012). However, many recent studies have suggested that this phenomenon is associated with neuronal inhibition (Shmuel et al. 2002, 2006; Smith et al. 2004; Pasley et al. 2007; Boorman et al. 2010; Wade and Rowland 2010; Mullinger et al. 2014; Sten et al. 2017) and also inhibition in the cerebro-cerebellar sensorimotor network (Hummel et al. 2004).

We found task-related deactivation across broad brain regions, including the ipsilateral (right) M1. The ipsilateral M1 deactivation was consistent between the active motor and passive proprioceptive unimanual tasks (Fig. 4*A*), corresponding with previous reports of ipsilateral M1 deactivation during unimanual motor tasks (Dettmers et al. 1995; Allison et al. 2000; Newton et al. 2005; Marchand et al. 2007; Hayashi et al. 2008) and ipsilateral SM1 deactivation during a nonmotor somatosensory stimulation task (Kastrup et al. 2008; Klingner et al. 2010; Schäfer et al. 2012; Gröschel et al. 2013; Klingner et al. 2015).

It is known that interhemispheric (transcallosal) inhibition exerted from the left to the right M1 can suppress superfluous activity in the right M1, and vice versa (Ferbert et al. 1992; Kobayashi et al. 2003). In older adults, reduced interhemispheric inhibition exerted from the contralateral M1 may contribute to age-related reduction/loss in ipsilateral M1 deactivation (Talelli et al. 2008), which is often reported during unimanual right-hand motor tasks (Hutchinson et al. 2002; Naccarato et al. 2006; Riecker et al. 2006; Ward et al. 2008; Loibl et al. 2011). These results, together with neurophysiological evidence from previous work (Palmer et al. 2012), support the presumption that interhemispheric inhibition exerted from the hand/arm section of the contralateral M1 contributes, at least partially, to the deactivation of the hand/arm section of the ipsilateral M1 in the present study. Therefore, its role might be to suppress ipsilateral activity during right-hand movements (Geffen et al. 1994), although we cannot exclude the possibility of inhibition from other brain structures (Blankenburg et al. 2003).

### Relationship Between Task-Related Deactivation and Hand/Finger Dexterity

Unlike active control-related activity in the contralateral M1, degree of task-related deactivation during the active and passive tasks in the hand/arm section of the ipsilateral M1 was correlated with the HDI only in the CH group (Fig. 4), meaning that the greater ipsilateral M1 deactivation during the active and passive tasks was related to greater dexterity only during childhood (Fig. 4*C*). We have recently shown that ipsilateral M1 deactivation during a right-hand unimanual motor task is increased from childhood to adolescence but stabilizes from adolescence to adulthood (Morita et al. 2019). Hence, the present results suggest that the correlation between ipsilateral M1 deactivation and greater dexterity (Fig. 4*C*) is confined to childhood, in which ipsilateral M1 deactivation and interhemispheric inhibition significantly develop. As shown in our previous study (Morita et al. 2019), we confirmed that ipsilateral M1 deactivation was greater in the AD group than in the CH group (Fig. 4*C*). In addition, the degree of ipsilateral M1 deactivation seemed to vary more in the AD group than in the CH group, indicating that its individual difference tends to be augmented through development (Fig. 4*C*).

Another important finding was that task-related M1 deactivation was correlated with greater dexterity during childhood in the trunk section of the ipsilateral M1 and the foot section of the contralateral M1 (Fig. 4*A*). Our previous study (Morita et al. 2019) also reported that cross-somatotopic deactivation increases from childhood to adulthood. The deactivation observed in the various somatotopic sections of the M1 in the current study (Fig. 4*A*) may help to functionally suppress the movement of irrelevant body parts during right hand movement, which has been suggested by previous studies (Jacobs and Donoghue 1991; Schneider et al. 2002). We speculate that the development of interhemispheric inhibition and cross-somatotopic inhibition might prevent other somatotopic sections from interfering with the hand/arm section of the contralateral M1 during right hand movements. Since these inhibitory functions appear to develop markedly during childhood (Morita et al. 2019), M1 deactivation in the ipsilateral hand/arm section and other somatotopic sections might serve as a determinant for greater hand/finger dexterity during childhood. Finally, as shown in Figure 4*C*, ipsilateral M1 deactivation in the hand/arm section may determine greater dexterity in children, in addition to active control-related activity in the hand/arm section of the contralateral M1 (Fig. 3*C*). However, these 2 factors seem to be independent, as indicated by a lack of significant correlation within children (*r* = 0.28).

In nonhuman primates, motor neurons in lamina IX of the spinal cord primarily receive efferents from the contralateral M1. Although the ipsilateral M1 also projects to the spinal cord, there are fewer terminals in lamina IX (Dum and Strick 1996; Morecraft et al. 2013). This indicates that a fast, direct corticomotor pathway that enables fine, dexterous, independent finger movements (Isa 2019; Lemon 2019) primarily originates from the contralateral M1, especially the new M1; that is, the caudal region of the M1 that likely corresponds to the human area 4p (Geyer et al. 1996; Rathelot and Strick 2009). The ipsilateral M1 spinal cord projections may terminate at spinal interneurons, which cannot directly control hand/finger muscles. The present findings in the CH group suggest that the brain’s fine control of dexterous hand/finger movements is largely derived through maturation of the contralateral pathway, whereas involvement of the ipsilateral pathway is reduced but can be recruited in case of brain stroke (Weiller et al. 1993; Jang et al. 2004; Lotze et al. 2006). Stroke patients with ipsilateral M1 activity often show a lack of dexterity. Similarly, children and elderly people with either less deactivation or a loss of deactivation in the ipsilateral M1 exhibit reduced dexterity (Fig. 4*C*; Loibl et al. 2011; Davidson and Tremblay 2013). This implies that reduction or loss of ipsilateral M1 deactivation (inhibition) may be an indicator for decline in hand/finger dexterity.

Using 2 dexterous motor tasks, the current work shows that human hand/finger dexterity generally develops from childhood to adulthood. We have further demonstrated, for the first time, that efficient recruitment of activity in the hand/arm section of the contralateral M1 during active hand motor control predicts greater hand/finger dexterity throughout childhood and into adulthood. Further, the degree of ipsilateral M1 inhibition also predicts hand/finger dexterity during childhood. The present study makes a significant contribution to the system neuroscience literature by highlighting the important role of the M1, an executive locus of motor control, in the maturation of human hand/finger dexterity.

## Notes

The authors are grateful to Dr Nobutsuna Endo, Ms Chie Kawakami, Ms Nodoka Kimura, and the CiNet technical staff for their support during the study. We would like to thank Editage (www.editage.com) for English language editing. *Conflict of Interest*: None declared.

## Funding

Grant-in-Aid for Scientific Research on Innovative Areas “Hyper-adaptation” (JSPS KAKENHI grant JP19H05723); Grant-in-Aid for Scientific Research (B) (JSPS KAKENHI grant: JP17H02143 to E.N., grant: JP20H04492 to T.M.). The funding sources had no involvement in the study design; in the collection, analysis, and interpretation of data; in the writing of the report; and in the decision to submit the article for publication.

## Credit Authorship Contribution Statement

E.N.: conceptualization, investigation, writing—review & editing, project administration, funding acquisition. T.M.: conceptualization, investigation, visualization, formal analysis, writing—original draft, funding acquisition. M.A.: writing—review & editing, supervision, funding acquisition.

## Supplementary Material

ActivePassive_PEG_CC_Supple_1104Final_tgaa085

Supplementary_video1_tgaa085

Supplementary_video2_tgaa085
