# Supplementary material for: Importance of the Primary Motor Cortex in Development of Human Hand/Finger Dexterity
Source: Cereb Cortex Commun. 2020 Dec 2;1(1):tgaa085. doi: 10.1093/texcom/tgaa085 (PMC8152843; doi:10.1093/texcom/tgaa085)
Supplement: ActivePassive_PEG_CC_Supple_1104Final_tgaa085 [file activepassive_peg_cc_supple_1104final_tgaa085.docx]

**Supplementary Material**

***An example of behavioral data during active and passive tasks***

We could not collect behavioral data during scanning. However, we collected goniometer data when a participant executed the active movements (active task) and experienced the passive movements performed by EN (passive task) later outside the scanner. We attached a goniometer (ShapeSensor S700, Measureland Inc., Fredericton, NB, Canada) to the participant’s right wrist. We collected the data at 1 kHz using a recording system (PowerLab 8/35, ADInstruments Ltd., New South Wales, Australia), applied a low-pass filter (2 Hz), and displayed the data using specialized software (LabChart 7 ADInstruments Ltd., New South Wales, Australia). Supplementary Figure 1 represents the goniometer data of right wrist movements for 15 sec (one epoch) during the active and passive tasks.

**Supplementary Figure 1.** Goniometer data of right wrist movements for 15 sec (one epoch) during the active (upper) and the passive (lower) tasks. Abbreviations: deg, degree

***EMG assessment outside the MRI scanner***

We could not record electromyograms (EMGs) during scanning. However, later, outside the scanner, we recorded EMGs when six right-handed healthy male adults (mean age 22.5 ± 1.26 years) executed the 1-Hz active movements (active task) and experienced the passive movements performed by EN (passive task). The participants were newly recruited from local universities. The participants were lying on a bed in a supine position and remained relaxed. The right forearm was placed over a cushion in order for it to be fully relaxed. The right hand was placed and fixed on the same apparatus (Figure 1B). Each participant performed the active and passive tasks in an alternating manner, with half of the participants starting with the active task. Each participant completed two experimental runs for each task. One run consisted of five task epochs, each of which lasted 15 s. Each participant started a run after he practiced the corresponding task for 15 s. During the rest period just before the participants started a run, participants were asked to keep the right hand in a straight (0°) position with the hand relaxed, and start with the flexion movement. When one epoch was finished, they had to keep the hand relaxed and in a straight position. Thus, the participants always started with the flexion movement in each epoch.

A surface EMG (sEMG) was recorded using an EMG system (Multi Analysis Programe MaP1038L, Nihonsanteku Co., Ltd., Osaka, Japan) at 2048 Hz from the flexor carpi radialis longus (FCR) and the extensor carpi radialis (ECR). A pair of bipolar Ag-AgCl electrodes with a 1 cm inter-electrode distance was attached on the skin surface of each muscle. An accelerometer (Map2466A-20G/1S, Nihonsanteku Co., Ltd., Osaka, Japan) was attached to the moving part of the apparatus, and its signal was also recorded in the system. We analyzed the data using this system. The raw sEMG signal was amplified with a gain of 1000, band-pass filtered at 10-500 Hz, and rectified. The accelerometer generated a signal when the hand touched the stopper (Figure 1B). An integrated EMG (iEMG) was calculated in each epoch for each muscle. The iEMG for the ECR was calculated for 14 s from the accelerometer signal when the hand made a first flexion contact with the stopper. Similarly, that for the FCR was calculated for 14 s from the signal when the hand made a first extension contact with the stopper. In so doing, we could evaluate 7 clusters of EMG activity for 14 s in each muscle. The data obtained from 10 epochs (5 epochs in two runs) were averaged and used for statistical analyses. The statistical analysis was performed using specialized software (IBM SPSS Statistics version 26). We conducted a two-way analysis of variance (ANOVA repeated measurement; task [2]: active, passive x muscle [2]: FCR, ECR).

We found robust EMG activity both in the FCR and in the ECR during the active task, while either no, very weak, or occasional activity was observed during the passive task. This was consistently observed in all participants. Supplementary Figure 2A demonstrates an example of EMG activity recorded from both muscles in an epoch during the active and passive tasks in a participant. As for the iEMG, the ANOVA showed significant main effects of task (F[1, 5] = 41.58, p = 0.001) and of muscle (F[1, 5] = 11.52, p = 0.019; Supplementary Figure 2B). Thus, EMG activity was significantly weaker during the passive task than during the active task, as shown in previous reports (Weiller et al. 1996; Jueptner et al. 1997). Hence, we may speculate that a similar phenomenon may have occurred during scanning.


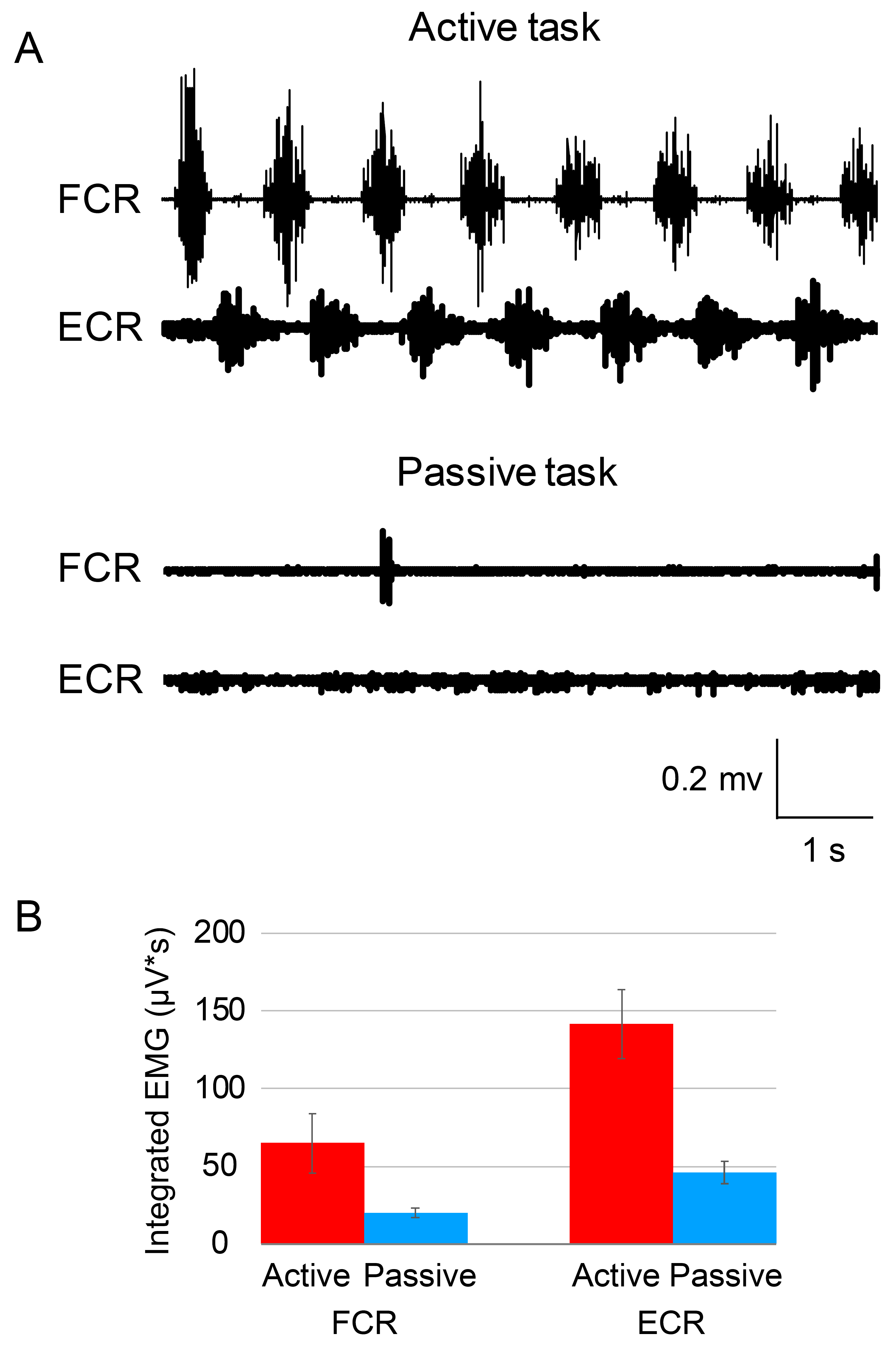


**Supplementary Figure 2.** EMG activity during active and passive tasks. (A) Examples of EMG activity recorded from the FCR and ECR during the active (upper panel) and passive (lower panel) tasks in a participant. (B) Integrated EMG results. The error bars reflect the SEM. Abbreviations: ECR, extensor carpi radialis; EMG, electromyogram; FCR, flexor carpi radialis longus; SEM, standard error of the mean

***Relationship between contralateral M1 activity during the passive task and that during the active task***

We carefully examined the relationship between the contralateral M1 activity during the passive task and that during the active task in all participants. For each participant, we extracted the effect size of task-related activity from the 4-mm (radius) sphere around the left M1 peak (-28, -28, 68) during the passive task or the active task separately. We then examined the correlations between the activity during the passive task and the active task across all participants (Supplementary Figure 3). We found that the activity in this region increased during the active task in all participants, and that this activity was highly positively correlated with that during the passive task [r(44) = 0.69]. We identified 26 participants who exhibited a robust increase of active control-related activity in the contralateral M1 region (active > passive), and 18 participants who did not show an increase in active control-related activity in this region (passive > active).





**Supplementary Figure 3.** Relationship between task-related activity in the contralateral (left) M1 region during the passive task and that during the active task. Each dot represents the individual measurement obtained from the 4-mm sphere around the left M1 peak (-28, -28, 68; see Figure 3C). The horizontal (x) and vertical (y) axes indicate effect size of the left M1 activity during the passive task (a.u.), and that during the active task (a.u.), respectively. The dashed line indicates y = x. The 18 participants below this line are those who did not show an increase in active control-related activity (passive > active). The 26 participants above this line are those who exhibited a clear increase in active control-related activity (active > passive). Abbreviations: a.u., arbitrary unit; M1, primary motor cortex.

**Characteristics of task-related activity during the passive task in the 18 participants**

To explore the brain regions that showed greater activity during the passive task in the 18 participants compared to the 26 remaining participants, we performed a two-sample t-test. We report group differences using the family-wise error rate (FWE)-corrected extent threshold of *p* < 0.05 across the entire brain for a voxel-cluster image generated at the uncorrected height threshold of *p* < 0.005. We found that in the whole brain, the sensorimotor network of area 2 (peak coordinates = -20, -44, 58; T = 4.84), caudal cingulate motor area (CMAc, 0, -20, 54; T = 4.14) and cerebellar vermis (4, -52, -2; T = 3.80), which all likely receive proprioceptive input and engage proprioceptive processing (Naito et al. 2016), were significantly more activated in the 18 participants than in the remaining 26 (Supplementary Figure 4). In this comparison, we did not find the contralateral M1 activity. The lack of contralateral M1 activity seems to rebuff the notion that the 18 participants generated a greater amount of active or resistant motor components during the passive task than the 26 participants.

We also conducted the same between-group comparison for task-related activity during the active task. However, we could not find any regions that exhibited significant differences. Hence, the results support our view that the 18 participants received a greater amount of proprioceptive input during the passive task (probably due to a higher sensory gain), even though all of the participants experienced the same proprioceptive task.

**Supplementary Figure 4.** Brain regions that showed greater activity during the passive task in the 18 participants compared to the 26 remaining participants. Data from horizontal and sagittal sections (z = + 58, x = - 3 and + 3) are shown.

**Repeatability of the present contralateral M1 finding**

To confirm the repeatability of the finding that reduced active control-related activity in the contralateral M1 is correlated with greater hand/finger dexterity (Figure 3), we conducted another fMRI study. A total of 32 healthy, right-handed adults (11 males; mean age, 34.7 ± 10.1 years; range, 25–59 years) participated in this study. This study included the same active and passive right-hand extension-flexion tasks, in addition to motor tasks with other body parts (foot, mouth, abdomen, and bimanual). To evaluate hand/finger dexterity, we used the same 12-hole peg task only.

The fMRI data acquisition parameters differed from those of the main study as follows: Functional images were acquired using T2*-weighted gradient echo echo-planar imaging (EPI) sequences obtained using a 3.0-Tesla MRI machine (Trio Tim; Siemens Healthineers, Erlangen, Germany) and a 32-channel array coil. We used a multiband imaging technique (multiband factor = 3). Each volume consisted of 48 slices across the entire brain acquired in an interleaved manner, with a slice thickness of 3.0 mm. The time interval between two successive acquisitions from the same slice (TR) was 1,000 ms. Echo time (TE) was 27 ms, and flip angle (FA) was 60º. The field of view (FOV) was 192 mm × 192 mm, and matrix size was 64 × 64. Voxel dimensions were 3 mm × 3 mm × 3 mm in the x-, y-, z-axes. Each participant completed one experimental 160-s run for each task. One run comprised 5 task epochs, each of which lasted for 15 s. The task epochs were separated by 15-s baseline (rest) periods. We collected 160 volumes in one experimental run.

As in the main study, active control-related activity was evaluated for each participant by subtracting activity during the passive task from that during the active task (active – passive). We calculated individual HDIs based on the individual best (shortest) time for the three trials of the 12-hole peg task. We calculated z-scores of individual performance based on the mean and its standard deviation across all 32 participants. The sign of the z-score was reversed, so that higher z-scores represented better performance.

We conducted a regression analysis using individual images showing active control-related activity. In this analysis, we only included a regressor of the HDI, since we found no significant correlation [r(32) = 0.19] between the HDI and age across participants.

When we generated a voxel-cluster image with an uncorrected height threshold of *p <* 0.001, we found a significant voxel cluster in the contralateral M1/PMD (voxel size = 158; FWE-corrected extent threshold of *p* < 0.05 across the entire brain), in which active control-related activity was negatively correlated with the HDI. This was the largest cluster in the entire brain. We further conducted ROI analyses [using a small volume correction (SVC) approach] to examine whether the active control-related activity in the contralateral M1 regions (peak coordinates = [-28, -28, 68] and [-24, -26, 56]) that were identified in the main study (Figure 3B) was also negatively correlated with the HDI in this independent adult group. The ROI was a 12-mm (radius) sphere around each M1 peak. We found a significant cluster (13 voxels; *p* = 0.02 after SVC) in the ROI around the latter area 4p peak (-24, -26, 56).

These results supported the repeatability of the current findings, i.e., reduced active control-related activity in the contralateral M1 identified by the active and passive tasks is correlated with greater hand/finger dexterity (Figure 3), and further indicated that such a relationship could be extended to a wider age range of adults.
